# EEG Spectral Feature Modulations Associated With Fatigue in Robot-Mediated Upper Limb Gross and Fine Motor Interactions

**DOI:** 10.3389/fnbot.2021.788494

**Published:** 2022-01-20

**Authors:** Udeshika C. Dissanayake, Volker Steuber, Farshid Amirabdollahian

**Affiliations:** School of Physics, Engineering and Computer Science, University of Hertfordshire, Hatfield, United Kingdom

**Keywords:** electroencephalogram, fatigue in upper limb robot-mediated interactions, HapticMASTER, SCRIPT passive orthosis, relative band power, band power ratios, independent component analysis, statistical analysis

## Abstract

This paper investigates the EEG spectral feature modulations associated with fatigue induced by robot-mediated upper limb gross and fine motor interactions. Twenty healthy participants were randomly assigned to perform a gross motor interaction with HapticMASTER or a fine motor interaction with SCRIPT passive orthosis for 20 min or until volitional fatigue. Relative and ratio band power measures were estimated from the EEG data recorded before and after the robot-mediated interactions. Paired-samples *t*-tests found a significant increase in the relative alpha band power and a significant decrease in the relative delta band power due to the fatigue induced by the robot-mediated gross and fine motor interactions. The gross motor task also significantly increased the (θ + α)/β and α/β ratio band power measures, whereas the fine motor task increased the relative theta band power. Furthermore, the robot-mediated gross movements mostly changed the EEG activity around the central and parietal brain regions, whereas the fine movements mostly changed the EEG activity around the frontopolar and central brain regions. The subjective ratings suggest that the gross motor task may have induced physical fatigue, whereas the fine motor task may have induced mental fatigue. Therefore, findings affirm that changes to localised brain activity patterns indicate fatigue developed from the robot-mediated interactions. It can also be concluded that the regional differences in the prominent EEG spectral features are most likely due to the differences in the nature of the task (fine/gross motor and distal/proximal upper limb) that may have differently altered an individual's physical and mental fatigue level. The findings could potentially be used in future to detect and moderate fatigue during robot-mediated post-stroke therapies.

## 1. Introduction

Fatigue experienced during post-stroke upper limb rehabilitation and its implications for the therapy outcome are often overlooked in existing therapy sessions. Many stroke survivors (about 30 to 70%) have reported persistence of fatigue as a debilitating symptom (Staub and Bogousslavsky, 2001; Lerdal et al., 2009). It is more likely that the increased motor/cognitive processing demands required during motor retraining exercises may exacerbate stroke patients' fatigue levels. The elevated fatigue levels may impair motivation and compliance to effectively perform the therapeutic interactions and the long-term commitment toward rehabilitation. Furthermore, some studies have reported that high-intensity fatiguing tasks are detrimental to both motor performance and learning (Godwin and Schmidt, 1971; Carron, 1972; Thomas et al., 1975; Williams and Singer, 1975; Branscheidt et al., 2019), whereas some investigations have only found performance impairments (Alderman, 1965; Carron, 1969; Cotten et al., 1972). Sterr and Furlan (2015) hypothesised that the relationship between training intensity and motor performance of constraint-induced therapy in chronic hemiparetic stroke patients is modulated by fatigue in addition to the residual motor ability. Foong et al. (2019) also suggested that the poor performance in the nBETTER (Neurostyle Brain Exercise Therapy Towards Enhanced Recovery) system could be due to the mental fatigue that progressed during the therapy. In Prasad's et al. (2010) study where chronic hemiplegic stroke patients performed both physical practice and motor imagery, a trend of more considerable variability in the brain-computer interface (BCI) performance was observed with the rise in individual fatigue levels. Therefore, it is highly questionable whether continuing a stroke therapy while or beyond fatigued conditions would impede motor performance and motor skill relearning during therapeutic interactions.

Despite its clinical importance, there exists no unambiguous and universally agreed definition for the term fatigue. In general, fatigue is a sensation of tiredness, weariness or lack of energy that is experienced following or during prolonged physical or mental activity. Fatigue can be broadly categorised into two types: physical (or muscular) fatigue and mental fatigue. Physical fatigue is defined as a failure to maintain force (or power output) during sustained muscle contractions (Gibson and Edwards, 1985). In contrast, mental fatigue is a subjective feeling of tiredness experienced during or after prolonged periods of demanding cognitive activity (Lorist et al., 2005). Recent studies have also shown that mental fatigue impairs physical performance, especially in sports-related activities (Marcora et al., 2009; Mehta and Parasuraman, 2014; Van Cutsem et al., 2017). Electroencephalogram (EEG) has shown to be the most predictive and promising biomarker of fatigue (Lal and Craig, 2001; Tran et al., 2020). To date, many studies have investigated EEG feature modulations associated with fatigue, including fatigue induced by driving tasks (Lal and Craig, 2002; Eoh et al., 2005; Tran et al., 2008; Jap et al., 2009; Craig et al., 2012; Zhao et al., 2012; Borghini et al., 2014), voluntary motor tasks (Yao et al., 2009; Wang et al., 2017), cognitive tasks (Massar et al., 2010; Tanaka et al., 2012; Trejo et al., 2015), brain-computer interfaces (Käthner et al., 2014), exercises and sports-related activities (Bailey et al., 2008; Barwick et al., 2012; Baumeister et al., 2012; Xu et al., 2018), visual display terminal tasks (Cheng and Hsu, 2011; Fan et al., 2015). However, the alterations in EEG activity caused by fatigue accumulated following robot-mediated interactions have not yet been comprehensively explored to the author's knowledge. EEG-based fatigue indices could be used to mitigate fatigue accumulated during human-robot interactions, thereby enhancing the efficacy of rehabilitation and reducing fatigue-related risks in human-robot collaboration tasks.

EEG consists of a wide frequency spectrum, and spectral features (band power and band power ratios) are frequently used as indicators of fatigue. Table 1 summarises the findings of 16 studies over the last two decades identified by a systematic review on EEG spectral feature modulations caused by fatigue. It was evident that in most studies, θ and α band power increased and β band power decreased significantly as a result of fatigue (Lal and Craig, 2002; Eoh et al., 2005; Barwick et al., 2012; Craig et al., 2012; Zhao et al., 2012; Käthner et al., 2014; Fan et al., 2015; Trejo et al., 2015; Zou et al., 2015; Wang et al., 2017; Xu et al., 2018). Some studies investigated the variations in delta band power as well; however, not many studies were able to identify significant variations with fatigue (Caldwell et al., 2002; Lal and Craig, 2002; Jap et al., 2009; Craig et al., 2012; Tanaka et al., 2012; Zhao et al., 2012; Chen et al., 2013; Fan et al., 2015). In these studies, EEG band power is given as absolute band power or relative band power. The relative band power is defined as a ratio between the absolute band power of each frequency band and the total power of all frequency bands in consideration. EEG band power ratios: (θ + α)/β, α/β, (θ + α)/(α + β), and θ/β were also used in some studies since the basic band powers can be insufficient to observe the shift of brain activity from fast waves to slow waves (Eoh et al., 2005; Jap et al., 2009; Fan et al., 2015). EEG band power ratios showed a significant increase with fatigue build-up. Eoh et al. (2005) stated that the index (θ + α)/β was a more reliable fatigue indicator during a simulated driving task due to the mutual addition of α and θ activity during the repetitive phase transition between wakefulness and microsleep. Jap et al. (2009) also reported a greater increase in the index (θ + α)/β, in comparison to the other power ratios, when a person experienced a fatigued state at the end of a monotonous simulated driving task. Most studies have also found a widespread topographical distribution in the changes in EEG spectral features with fatigue. However, some studies are equivocal and need further exploration (Jap et al., 2009; Cheng and Hsu, 2011; Baumeister et al., 2012; Tanaka et al., 2012; Chen et al., 2013). Variations in methodological approaches, including low sample size, differences in the fatiguing study protocol, the number of electrodes used, the electrode placement and the feature definition, could be a possible explanation for the discrepancies across the studies.

**Table 1:** Literature summary on modulations in the EEG spectral features with fatigue.

**Reference**	**Description**	**No of participants**	**No of electrodes**	**δ**	**θ**	**α**	**β**	** $\frac{\left(\theta +\alpha \right)}{\beta }$ **	** $\frac{\alpha }{\beta }$ **	** $\frac{\left(\theta +\alpha \right)}{\left(\alpha +\beta \right)}$ **	** $\frac{\theta }{\beta }$ **	**Electrode locations or brain regions modulated by fatigue**
Barwick et al. (2012)	Fatigue during administration of a neuropsychological test battery	14	42	-	↑^*R*^	↑^*R*^	↓^*R*^	-	-	-	-	F, C, P, O
Baumeister et al. (2012)	Effects of fatigue induced by a cycling exercise on knee joint reproduction task	12	22	-	↓	↓^*L, U*^	-	-	-	-	-	F3, Fz, F4, FC3, FCz, FC4, P4, O1, Oz, O2, T5
Chen et al. (2013)	Fatigue induced by watching 3DTV	10	16	↑^*R*^	NS	↓^*R*^	↓^*R*^	↑	↑	↑	↑	FP1, FP2, F3, C3, C4, F7, F8, T5
Cheng and Hsu (2011)	Mental fatigue induced by visual display terminal tasks	20	7	-	↑^*R*^	↓^*R*^	NS	↓	NS^a^	-	-	F3, Fz, F4, Cz, Pz, O1, O2
Craig et al. (2012)	Fatigue induced by monotonous simulated driving task	48	32	NS	↑	↑^*L, U*^	↑	-	-	-	-	FL, FM, FR, CL, CM, CR, POL, POM, POR
Eoh et al. (2005)	Fatigue during a simulated driving task	8	8	-	NS	↑^*R*^	↓^*R*^	↑	↑^*a*^	-	-	
Fan et al. (2015)	Mental fatigue in visual search task	10	64	NS	NS	↑^*R*^	↓^*R*^	↑	↑	↑	↑	FP, IF, F, C, P, O, T, PT
Jap et al. (2009)	Fatigue induced during a monotonous driving session	52	30	*	*	↓	↓	↑	↑	↑	↑	F, C, P, T, EB
Käthner et al. (2014)	Mental fatigue during P300 brain computer interface	12	31	-	↑	↑	-	-	-	-	-	F3, Fz, F4, FC5, FC3, FCz, FC4, FC6, C5, C3, Cz, C4, C6, CP5, CP3, CPz, CP4, CP6, P3, P1, Pz, P2, P4, PO7, PO3, POz, PO4, PO8, O1, O2
Lal and Craig (2002)	Fatigue during simulated driving task	35	19	↑	↑	↑	↑	-	-	-	-	EB
Tanaka et al. (2012)	Mental fatigue induced by 0^(NS)^ or 2-back test	18	11	NS	↑	↓	↓	-	-	-	↑	Fz, P3, Pz, O1, O2
Trejo et al. (2015)	Mental fatigue induced by a sustained low-workload mental arithmetic task	16	2	-	↑	↑	-	-	-	-	-	Fz, Pz
Wang et al. (2017)	Muscle fatigue during right arm side lateral raise task with loads	18	2	-	-	↑	NS	-	-	-	-	C3, C4
Xu et al. (2018)	Fatigue in mental^(NS)^ and physical-mental task	14	16	-	-	-	↓^*R*^	-	↑	-	-	C3, P3, Pz, Oz, T3, T4, T5
Zhao et al. (2012)	Mental fatigue in simulated driving task	13	32	NS	↑^*R*^	↑^*R*^	↓^*R*^	-	-	-	-	F, C, P, O, T
Zou et al. (2015)	Stereoscopic 3D visual fatigue caused by vergence-accommodation conflict	11	30	-	NS	↑^*R*^	↓^*R*^	*	*	NS	NS	F, C, P, EB

↑ = significant increase; ↓ = significant decrease; * = significant, but the direction of change is not specified; NS = no significant change; - = not reported; R = relative band power measures were considered; L, U = lower and upper bands were considered; a = β/α was reported; The brain regions denoted by FP, IF, F, FL, FM, FR, FC, C, CL, CM, CR, P, PO, O, T, PT, POL, POM, POR, and EB corresponds to frontopolar (or pre-frontal), inferior frontal, frontal, left frontal, midline frontal, right frontal, fronto-central, central, central left, midline central, central right, parietal, parieto-occipital, occipital, temporal, posterior temporal, posterior left, midline posterior, posterior right and entire brain average.

The type of fatigue experienced during robot-mediated exercises may depend on the exercise mode, intensity and condition of the patient. For instance, the upper limb joints and muscles involved in an interaction may vary from one therapy to another depending on the severity of the impairment of fine or gross motor skills. Gross motor skill retraining exercises such as arm reach/return exercises are primarily involved in the movement and coordination of proximal joints and muscles of the upper limb (shoulder and arm). In contrast, fine motor skill retraining exercises involve coordination of the distal joints and muscles of the upper limb (hand, wrist, and fingers). Cowley and Gates (2017) found that proximal fatigue in a repetitive, timed movement task significantly changes the movement in trunk shoulder and elbow kinematics, whereas the changes were mainly in wrist and hand movement due to distal muscle fatigue. Therefore, in general, repetitive gross motor skill retraining exercises may induce more physical fatigue than fine motor skill retraining exercises. In addition, most therapeutic fine motor activities require considerable attention and decision-making skills combined with hand, wrist and finger movements; therefore, they may induce more mental fatigue than most gross motor exercises. As the type of prominent fatigue developed during a robot-mediated interaction may vary depending on the physical and mental workload associated with the task, cortical sites that show significant variations in EEG spectral features following fatigue may differ between interactions. However, these differences between gross and fine motor robot-mediated interactions are not systematically investigated.

In this preliminary experiment, we hypothesised that the EEG correlates of fatigue induced by robot-mediated interactions are specific to the physical or cognitive nature of the task and the differences in the usage of the proximal or distal upper limb. The gross movements (arm reach/return) were performed using the HapticMASTER (Motekforce Link, The Netherland) (Amirabdollahian et al., 2007; Chemuturi et al., 2013), and the fine movements (hand open/close) were performed using the SCRIPT passive orthosis (Amirabdollahian et al., 2014). Given the differences in the two tasks, it could be expected that the gross motor task may induce more physical fatigue than the fine motor task, in which more mental fatigue may be visible. Therefore, it was anticipated that the resulting statistically significant differences in EEG spectral features might show varying topographical distributions between the two robot-mediated interactions. Furthermore, significant changes to the EEG spectral features localised around the motor cortex were expected following the robot-mediated gross movements since fatigue may affect motor coordination skills. In the fine motor robot-mediated interaction that requires more attention and decision making, significant changes to the frontopolar brain activities were expected in addition to the attenuation in the activities around the motor cortex.

## 2. Materials and Methods

### 2.1. Ethical Approval

The experiment was approved by the Science and Technology Ethics Committee with Delegated Authority of the University of Hertfordshire (Protocol numbers: COM/PG/UH/00100 and aCOM/PG/UH/00100).

### 2.2. Participants

Twenty healthy right-handed volunteers, who were at least 20 years of age (average age of the sample was 32 ± 10 years; mean ± SD) and with no history of severe injury to the head, brain, or right hand participated in this experiment. Right-handedness was considered since both robotic interfaces were constrained to be used only by the right upper limb due to their hardware configurations and setup. All participants had normal vision or corrected to normal vision. All participants signed informed consent forms before participation.

### 2.3. Fatigue Inducing Robot-Mediated Interactions

**Figure 1:**
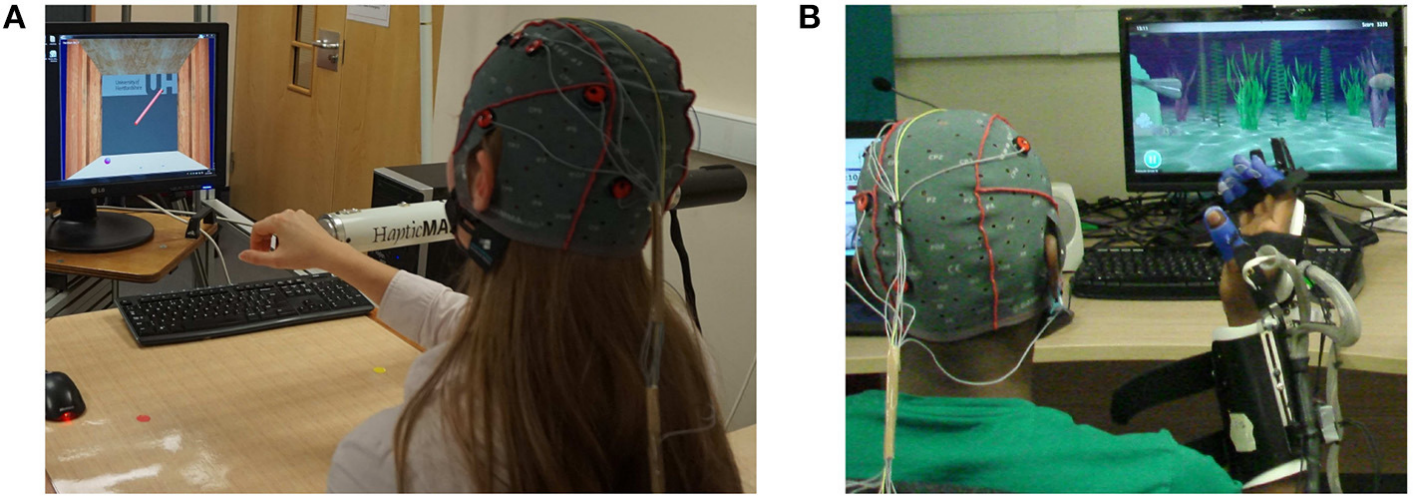
Fatigue inducing robot-mediated interactions. **(A)** Robot-mediated gross motor interaction (arm reach/return task) using HapticMASTER, and **(B)** robot-mediated fine motor interaction (hand open/close task) using SCRIPT passive orthosis.

Given the consent to take part in the experiment, participants were randomly assigned into two groups: A and B, with 10 participants in each group. Participants in group A performed visually guided arm reach/return movements with HapticMASTER (gross motor task, Figure 1A), whereas participants in group B performed hand open/close movements with SCRIPT passive orthosis (fine motor task, Figure 1B). Both robot-mediated interactions were performed for 20-min or until volitional fatigue. The virtual reality environment of the GENTLE/A rehabilitation system (Chemuturi et al., 2013) was used for the gross motor task. Target point locations were modified so that the trajectory covered by the movement of the HapticMASTER robot arm was mapped into a straight line connecting only two virtual target points. In addition, the HapticMASTER was set to active mode so that the participants should initiate the movement and reach the target points by themselves. The virtual reality game “sea shell,” developed for the SCRIPT system, was used as the fine motor task (Amirabdollahian et al., 2014). Participants performed hand open/close gestures to open/close a seashell underwater to catch a fish near the seashell. Both robot-mediated interactions were performed using only the right hand, and participants were asked to keep their left hand in a relaxed position throughout the task. The distance between the computer monitor and the participant's eye was set to around 120 cm for both groups.

### 2.4. EEG Data Acquisition

**Figure 2:**
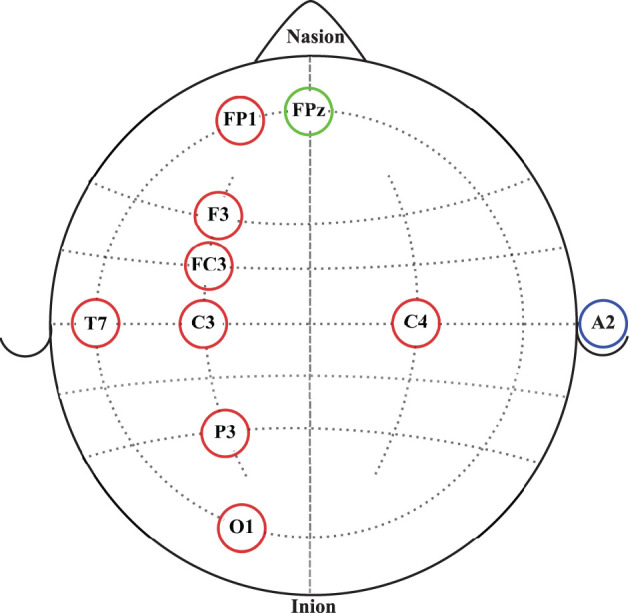
EEG electrode placement according to the International 10-10 system of electrode placement. Red circles represent the eight active electrodes selected for the data acquisition. The blue circle represents the reference electrode location. The green circle represents the ground electrode location.

Continuous EEG signals were recorded before, during and after the robotic interactions using an eight-channel EEG data acquisition system, g.MOBIlab+ (g.tec medical engineering GmbH, Austria) with active electrodes. According to the International 10-10 system of electrode placement (American, 2006), FP1, F3, FC3, C3, C4, P3, O1, and T7 electrode locations were selected as shown in Figure 2. All electrodes were referenced to the right earlobe (A2), and FPz was used as the ground electrode. Signals acquired by the active electrodes are pre-amplified directly at the electrode (Pinegger et al., 2016). Also, the active electrode system reduces or avoids artifacts caused by high impedance between the electrode(s) and the skin (e.g., 50/60 Hz coupling, electrode or cable movement artifacts, background noise) (g.tec medical engineering GmbH, 2014b). The sampling rate, lower and upper cut-off frequencies of the bandpass filter of the amplifier are fixed at 256, 0.5, and 100 Hz, respectively, by the manufacturer. Therefore, signals acquired from this device were sampled at 256 Hz and had a fixed EEG bandwidth of 0.5 to 100 Hz.

### 2.5. Experimental Procedure

**Figure 3:**
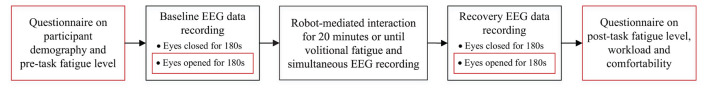
Flow diagram of the proposed experiment.

On arrival at the laboratory, participants were informed about the experiment protocol, given time to familiarise themselves with the assigned robotic interaction and were prepared for the EEG data collection according to the guidelines given in g.tec medical engineering GmbH (2014a). The flow diagram of the proposed experiment is given in Figure 3. Following the standardised EEG recording protocol, EEG data were recorded before, during and after the robot-mediated interactions. Participants were instructed to close and open their eyes for 180 s each when EEG data were recorded before and after the gross and fine motor tasks. In order to reduce artifacts in the EEG data recorded with eyes opened/closed, participants were instructed to sit still while minimising eye blinks, eye movements, swallowing, jaw clenching, or any other severe body movements. In this paper, only the EEG data recorded with eyes opened are further analysed. Participant's feedback on their physical and mental fatigue level before and after the tasks were obtained using two statements with a 5-point Likert rating scale (i.e., 1 = “Not at all fatigued,” 2 = “somewhat fatigued,” 3 = “moderately fatigued,” 4 = “very fatigued,” and 5 = “extremely fatigued”). Also, the participant's feedback on the task-associated physical and mental workload was obtained using two statements with a 5-point Likert rating scale (i.e., 1 = “Not at all demanding,” 2 = “somewhat demanding,” 3 = “moderately demanding,” 4 = “very demanding,” and 5 = “extremely demanding”) (Dissanayake, 2021). Moreover, all participants performed the assigned task for 20 min.

### 2.6. EEG Data Analysis

**Figure 4:**
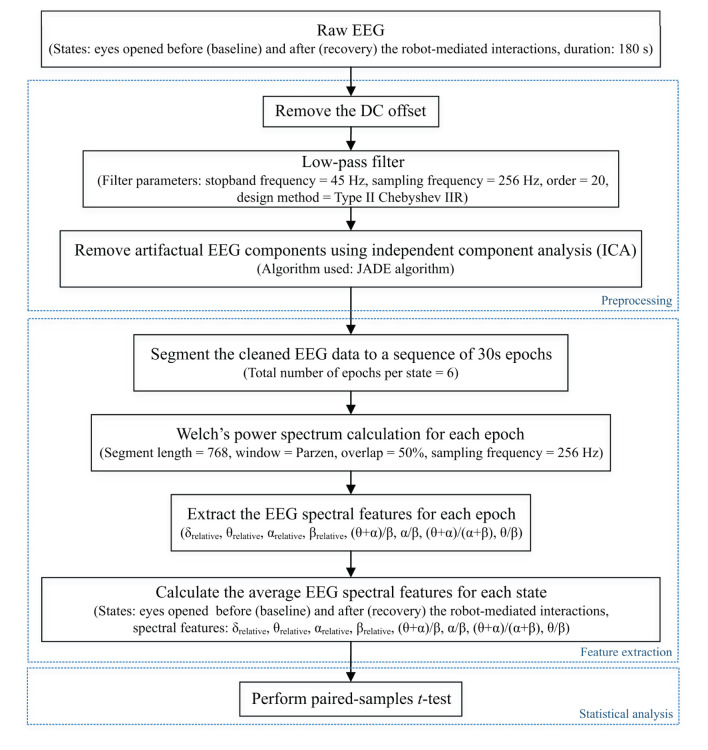
EEG data processing pipeline followed to preprocess raw EEG data and extract EEG spectral features of each state for each participant to perform the statistical analysis. Dotted boxes represent the three main steps involved in the pipeline: data preprocessing, feature extraction, and statistical analysis. δ_relative_, θ_relative_, α_relative_, and β_relative_ indicate the relative δ, θ, α and β band powers, respectively, and (θ + α)/β, α/β, (θ + α)/(α + β), and θ/β indicate the power ratios.

This paper reports the modulation of EEG spectral features during eyes opened states before and after the fatiguing robot-mediated interactions. EEG features extracted from the data recorded before the task is referred to as baseline, and the data recorded after the task is referred to as recovery, respectively, throughout this paper. These states can be considered to reflect the restfulness of the participant before and after the robotic interactions; thereby, any changes in these states could be a reflection of fatigue. Previous studies have also compared EEG data recorded before and after a task to identify EEG feature modulations associated with fatigue induced by physical and mental tasks (Ng and Raveendran, 2007; Cheng and Hsu, 2011; Tanaka et al., 2012; Chen et al., 2013). The EEG data processing pipeline followed for each participant during baseline and recovery states is illustrated in Figure 4. EEG preprocessing and feature extraction was performed offline using custom MATLAB scripts.

#### Preprocessing

Firstly, the DC offset of each recording was removed by subtracting the channel-wise mean from each data point. Then, a Type II Chebyshev low-pass filter with a stopband frequency of 45 Hz and an order of 20 was applied to eliminate the power line noise (50 Hz) distortions.

The EEG research community widely uses independent component analysis (ICA) to separate and remove artifacts in EEG signals (Makeig et al., 1996; Jung et al., 1998; Delorme et al., 2007; Debener et al., 2010). ICA is a linear decomposition technique used to recover a set of *n* unobserved independent source signals given only *m* ≥ *n* observed instantaneous mixtures of these source signals. If we denote the *n* independent source signals at time *t* by a *n* × 1 vector **s**(*t*) and the observed signals by a *m* × 1 vector **x**(*t*), the mixing model can be written as,


(1) $$\begin{array}{l} \text{x}\left(t\right)=\text{As}\left(t\right), \end{array}$$

where the *m* × *n* matrix **A** represents the unknown “mixing matrix”. The elements in each row of **A** corresponds to the contributions from each source signal to each observation (i.e., $x_{i}\left(t\right)=\overset{n}{\underset{j=1}{\sum }}a_{ij}s_{j}\left(t\right)$ for all *i* = 1 to *m*). The objective of ICA is to find a separating matrix, i.e., a *n* × *m* matrix **W** such that


(2) $$\begin{array}{l} \text{u}\left(t\right)=\text{Wx}\left(t\right) \end{array}$$

is an estimate of the original source signals. The elements in the *n* × 1 vector **u**(*t*) (i.e., independent components) are identical to the original source signals up to permutations and changes of scales and signs (Cardoso, 1998).

**Figure 5:**
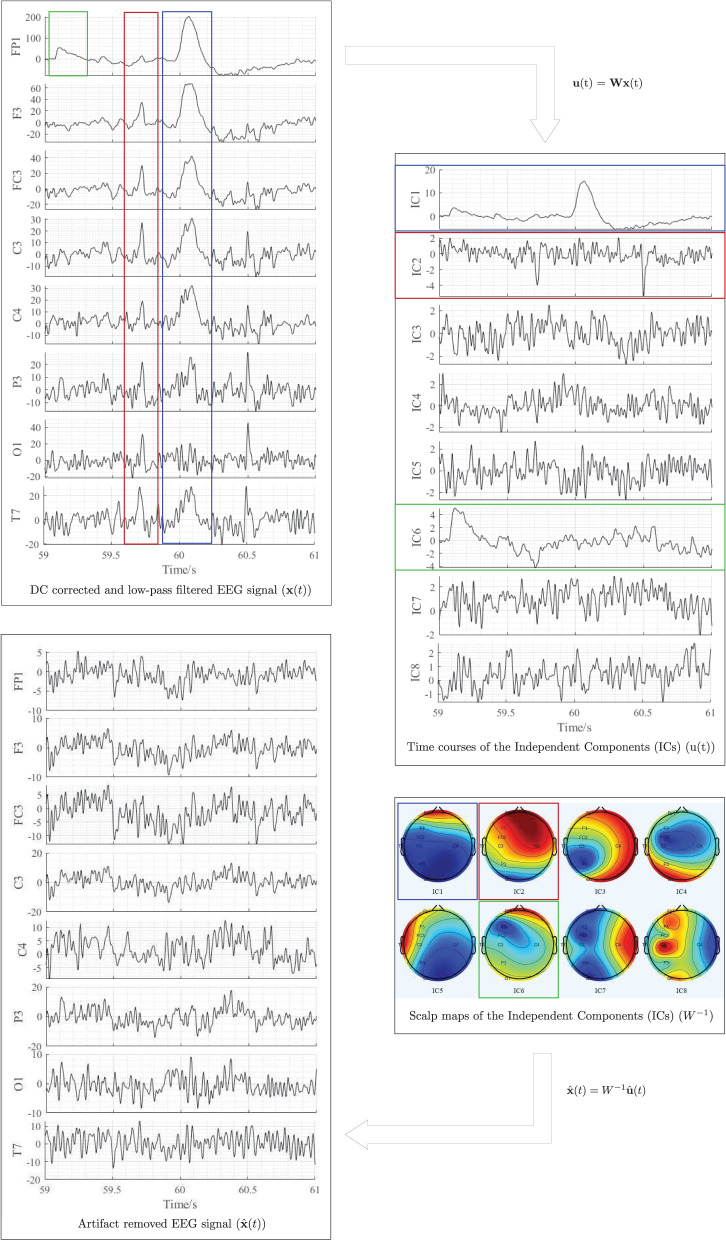
Illustration of EEG artifactual components identification using ICA for one participant. The red, blue, and green rectangles correspond to cardiac activity, eye blink, and eye movement artifacts, respectively. All data were referenced to the right earlobe (A2).

The joint approximate diagonalisation of eigenmatrices (JADE) algorithm (Cardoso and Souloumiac, 1993) was used in this experiment to separate and remove in-band artifacts, including eye blinking, eye movement, swallowing, jaw clenching, and cardiac activity from the independent components. Figure 5 illustrates the artifact removal process using ICA for a single subject. When applying ICA to separate EEG artifacts from brain activity patterns, it was assumed that the signals emitted by the unobserved sources are independent, and the number of independent sources is the same as the number of electrodes used in the experiment (i.e., *m* = *n* = 8). The relative projection strengths of each independent component onto the scalp electrodes were given by the columns of the inverse separation matrix **W**^−1^, which is an estimate of the mixing matrix **A** in Equation 1. The “corrected” EEG signal was then derived as, $\hat{\text{x}}\left(t\right)=W^{-1}\hat{\text{u}}\left(t\right)$, where $\hat{\text{u}}\left(t\right)$ was derived from the matrix of activation waveforms **u**(*t*), by setting the rows representing the artifactual components identified by visual inspection to zero (Jung et al., 2000).

#### Feature Extraction

The corrected EEG signals at the two states: baseline and recovery for each participant were segmented into epochs of 30 s length (i.e., 7680 samples per epoch and 6 epochs in total per state). The power spectral density for all epochs was estimated using Welch's averaged modified periodogram method (Welch, 1967) with a 3 s segment length (i.e., 768 samples), 50% overlap, and a Parzen window. Subsequently, the relative band power of δ (1- < 4 Hz), θ (4- <8 Hz), α (8–13 Hz), and β (<13–30 Hz) (denoted by δ_relative_, θ_relative_, α_relative_, and β_relative_, respectively, in this paper) for each epoch were calculated as a ratio between the average band power of each frequency band and the total band power (i.e., the summation of average δ, θ, α, and β band powers). The four ratio band power measures: (θ + α)/β, α/β, (θ + α)/(α + β), and θ/β for each epoch were also calculated. Finally, the average of each EEG spectral feature within the 180 s duration (i.e., six epochs) of each state was calculated to represent the corresponding spectral EEG feature index of the baseline and recovery states, respectively (Dissanayake, 2021).

#### Statistical Analysis

Statistical analysis was carried out using the *IBM SPSS Statistics 25* software. A *p*-value < 0.05 was considered statistically significant, denoting a 95% confidence interval. It was of interest to investigate whether the significant differences in EEG spectral features caused by fatigue are localised to different electrode locations due to the differences in the nature of the task (fine/gross motor and distal/proximal upper limb). Normality of the differences between EEG spectral features extracted from baseline and recovery states were assessed using the Kolmogorov-Smirnov test. Upon confirmation of normal distribution, two-tailed paired-samples *t*-tests were performed separately on the eight electrode locations to identify the significant differences between the baseline and recovery states of each EEG spectral feature for each robot-mediated interaction. The effect sizes were expressed by the Pearsons' correlation coefficient, $r=\sqrt{\frac{t^{2}}{\left(t^{2}+df^{2}\right)}}$. Multiple paired-samples *t*-tests were also used in previous fatigue studies to evaluate the changes in EEG features at different brain regions (Tanaka et al., 2012; Zhao et al., 2012; Chen et al., 2013; Fan et al., 2015).

## 3. Results

### 3.1. Modulations in EEG Spectral Features Following the Robot-Mediated Gross Motor Interaction With HapticMASTER

**Figure 6:**
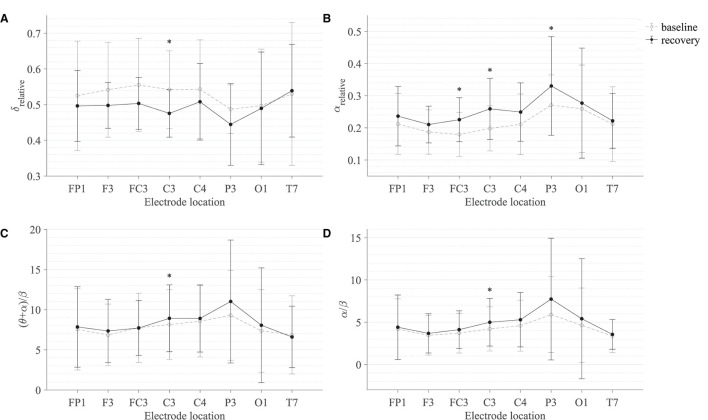
Comparison of the sample mean and standard deviation of **(A)** δ_relative_, **(B)** α_relative_, **(C)** (θ + α)/β, and **(D)** α/β of all participants between baseline and recovery states for the gross motor interaction with HapticMASTER. The statistical significance is represented by an asterisk: i.e., *p < 0.05.

**Figure 7:**
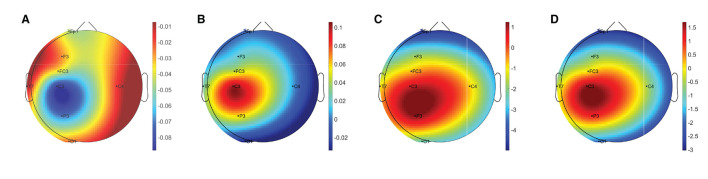
Brain topographies for the difference between recovery and baseline states (i.e., difference = recovery - baseline) of **(A)** δ_relative_, **(B)** α_relative_, **(C)** (θ + α)/β, and **(D)** α/β for one participant following the gross motor interaction with HapticMASTER. In each brain map, the nose is represented by the triangle on the top, and the right hemisphere is on the right. For α_relative_, (θ + α)/β, and α/β, the red-shaded areas indicate a larger increase whereas the blue-shaded areas indicate a decrease. For δ_relative_, the blue-shaded areas indicate a larger decrease whereas the red-shaded areas indicate a smaller decrease.

Table 2 summarises the paired-samples *t*-test results of the statistically significant EEG spectral feature modulations following the gross motor interaction with HapticMASTER. Figure 6 shows the sample mean and standard deviation of the substantive EEG spectral features during baseline and recovery states. Comparison of the sub-figures shows that α_relative_ changed the most due to fatigue induced by the gross motor interaction with HapticMASTER. In Figure 6B, there is a clear increase in the sample mean of α_relative_ across all electrodes, with statistically significant differences visible on the three electrodes placed over the contralateral motor cortex: FC3 (*t*(9) = -2.378, *p* = 0.041, *r* = 0.621), C3 (*t*(9) = -3.148, *p* = 0.012, *r* = 0.724) and P3 (*t*(9) = -2.646, *p* = 0.027, *r* = 0.661). As well as being statistically significant, the effect of the variation in α_relative_ on FC3, C3, and P3 electrodes is large. These electrodes correspond to motor activities using the right hand; thereby, the significant increase in α_relative_ reflects a decreased cortical activation, which is an indication of fatigue. Similarly, Figures 6C,D show that fatigue induced by the gross motor task significantly increased both (θ + α)/β (*t*(9) = -2.787, *p* = 0.021, *r* = 0.681) and α/β (*t*(9) = -2.403, *p* = 0.040, *r* = 0.625) on the C3 electrode. A larger effect size was also visible on the C3 electrode for both (θ + α)/β and α/β. These findings show that fatigue induced by gross movements increased the low-frequency power and decreased the fast wave activities on the C3 electrode, resulting in a significant difference when combined. In contrast, Figure 6A indicates that there has been a drop in δ_relative_ following the gross movements (except on T7). Also, a significant variation with larger effect was found on the C3 electrode (*t*(9) = 2.593, *p* = 0.029, *r* = 0.654). This result is somewhat counter-intuitive because previous studies have shown a significant increase or no change in delta activity as fatigue progressed; however, it is reasonable to assume that this inconsistency may be related to the differences in experimental protocols.There were no significant differences visible in θ_relative_, β_relative_, (θ + α)/(α + β), and θ/β due to fatigue induced by the gross motor task. Overall, these results show a reduced activation around the sensorimotor cortex due to fatigue induced by robot-mediated gross movements. Figure 7 shows the brain topographies of the difference between recovery and baseline states (i.e., difference = recovery - baseline) of δ_relative_, α_relative_, (θ + α)/β and α/β for one participant who reported a greater increase in physical fatigue than mental fatigue following the gross motor task. Topographical distributions also show that the fatigue induced by the gross movements may have altered the EEG activity around the left central and left parietal regions.

**Table 2:** Significant EEG spectral feature modulations and the corresponding electrode locations following the gross motor interaction with HapticMASTER.

**Spectral feature**	**Electrode location**	**Sample mean** **±std**		**Paired samples** ***t*****-test**				**Direction of change**
		**Baseline**	**Recovery**	***t***	***df***	***p*-value**	***r***	
δ_relative_	C3	0.542 ± 0.109	0.476 ± 0.067	2.593	9	0.029	0.654	↓
α_relative_	FC3	0.180 ± 0.068	0.225 ± 0.069	-2.378	9	0.041	0.621	↑
	C3	0.198 ± 0.070	0.259 ± 0.095	-3.148	9	0.012	0.724	↑
	P3	0.271 ± 0.094	0.330 ± 0.154	-2.646	9	0.027	0.661	↑
$\frac{\left(\theta +\alpha \right)}{\beta }$	C3	8.151 ± 4.349	8.923 ± 4.167	-2.787	9	0.021	0.681	↑
$\frac{\alpha }{\beta }$	C3	4.213 ± 2.612	4.997 ± 2.812	-2.403	9	0.040	0.625	↑

*↑ = significant increase. ↓ = significant decrease*.

### 3.2. Modulations in EEG Spectral Features Following the Robot-Mediated Fine Motor Interaction With SCRIPT Passive Orthosis

**Figure 8:**
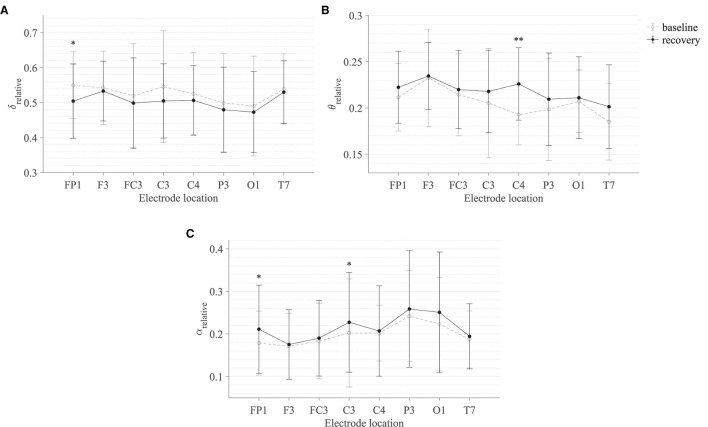
Comparison of the sample mean and standard deviation of **(A)** δ_relative_, **(B)** θ_relative_, and **(C)** α_relative_ of all participants between baseline and recovery states for the fine motor interaction with SCRIPT passive orthosis. The statistical significance is represented by an asterisk: i.e., *p < 0.05 and **p < 0.01.

**Figure 9:**
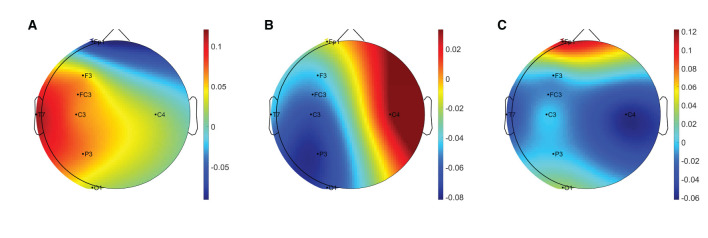
Brain topographies for the difference between recovery and baseline states (i.e., difference = recovery - baseline) of **(A)** δ_relative_, **(B)** θ_relative_, and **(C)** α_relative_ for one participant following the fine motor interaction with SCRIPT passive orthosis. In each brain map, the nose is represented by the triangle on the top, and the right hemisphere is on the right. The red-shaded areas indicate a larger increase whereas the blue-shaded areas indicate a larger decrease.

Table 3 summarises the paired-samples *t*-test results of the statistically significant EEG spectral feature modulations following the fine motor interaction with SCRIPT passive orthosis. Figure 8 shows the sample mean and standard deviation of the substantive EEG spectral features during baseline and recovery states. An increase of θ_relative_ and α_relative_ is visible in both Figures 8B,C on all electrodes. A significant increase in α_relative_ is visible on FP1 (*t* = -2.871, *p* = 0.018, *r* = 0.691) and C3 (*t* = -2.555, *p* = 0.031, *r* = 0.648) electrodes, whereas the significant difference in θ_relative_ is on the C4 electrode (*t* = -3.507, *p* = 0.007, *r* = 0.760). The effect of these significant variations in α_relative_ and θ_relative_ are also of larger magnitude, thereby suggesting that these variations are substantive findings. In contrast, a general decrease in δ_relative_ on all electrodes and a significant decrease on the FP1 electrode with a larger effect size (*t* = 3.066, *p* = 0.013, *r* = 0.715) can be found in Figure 8A. No significant differences were visible in β_relative_ and ratio band power measures. In general, these results show that the fatigue induced by fine motor interactions alters not only the activities around the sensorimotor cortex but also the frontopolar cortex. Figure 9 shows the brain topographies of the difference between recovery and baseline states (i.e., difference = recovery - baseline) of the substantive EEG features for one participant who reported a greater increase in mental fatigue than physical fatigue following the fine motor task. Topographical distributions also show that the variations in the EEG features around frontopolar and central brain regions may have been caused by the fatigue that resulted from the robot-mediated fine motor interaction.

**Table 3:** Significant EEG spectral feature modulations and the corresponding electrode locations following the fine motor interaction with SCRIPT passive orthosis.

**Spectral feature**	**Electrode location**	**Sample mean** **±std**		**Paired samples** ***t*****-test**				**Direction of change**
		**Baseline**	**Recovery**	***t***	***df***	***p*-value**	***r***	
δ_relative_	FP1	0.550 ± 0.096	0.504 ± 0.106	3.066	9	0.013	0.715	↓
θ_relative_	C4	0.193 ± 0.033	0.226 ± 0.039	-3.507	9	0.007	0.760	↑
α_relative_	FP1	0.179 ± 0.075	0.211 ± 0.104	-2.871	9	0.018	0.691	↑
	C3	0.202 ± 0.127	0.227 ± 0.117	-2.555	9	0.031	0.648	↑

*↑ = significant increase. ↓ = significant decrease*.

### 3.3. Subjective Measures of Fatigue Level and Workload

**Figure 10:**
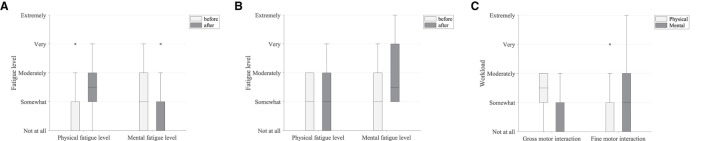
Subjective measures of fatigue level and workload. **(A)** Comparison of the subjective measures of fatigue level before and after the gross motor interaction with HapticMATER. **(B)** Comparison of the subjective measures of fatigue level before and after the fine motor interaction with SCRIPT passive orthosis. **(C)** Comparison of the subjective measures of physical and mental workload following the gross and fine motor interactions.

Most participants who performed the robot-mediated gross motor interaction with HapticMASTER reported increased physical fatigue following the task. Six participants showed a greater change in physical fatigue scores than in mental fatigue scores, and two participants showed an equal rise in both physical and mental fatigue scores. Therefore, the subjective ratings suggest that the gross motor interaction may have induced physical fatigue. In contrast, most participants who performed the fine motor task reported that their mental fatigue was increased following the robotic interaction. Four participants showed a greater change in mental fatigue scores than physical fatigue scores, and two participants showed an equal rise in both physical and mental fatigue scores. Therefore, the subjective ratings suggest that the fine motor interaction, on the other hand, may have induced mental fatigue. Furthermore, most participants reported that the gross motor task was more physically demanding than mentally demanding. In contrast, most participants revealed that the fine motor task required greater mental demand or equally physical and mental demand. A comparison of the subjective measures of physical and mental fatigue levels before and after the robot-mediated gross and fine motor interactions and the physical and mental workload of the two tasks is shown in Figure 10.

**Figure 11:**
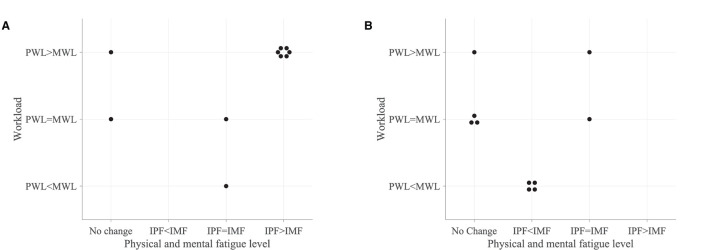
Association between the variations in fatigue levels and the rated workload following the **(A)** robot-mediated gross motor interaction with HapticMASTER and **(B)** robot-mediated fine motor interaction with SCRIPT passive orthosis. The “IPF” and “IMF” refers to the increase in physical and mental fatigue scores following the robot-mediated interactions, respectively. No change refers to no increase or a decrease in both fatigue levels. The “PWL” and “MWL” refers to the rated physical and mental workload, respectively.

Figure 11 shows the association between the variations in fatigue levels and the rated workload following the robot-mediated gross and fine motor interactions. All participants who experienced a greater increase in physical fatigue than the change in mental fatigue following the gross motor task also rated that the underlying physical workload of the gross motor task was greater than the mental workload. All participants who experienced a greater increase in mental fatigue than physical fatigue following the fine motor task rated that the fine motor task required a greater mental demand than the physical demand. The gross motor task involves the movement and coordination of proximal joints and muscles of the upper limb (shoulder and arm) to control the robot arm between target points. The fine motor task requires considerable attention and decision-making skills combined with hand and finger movements to catch the fish when it reaches the seashell. Therefore, the subjective responses imply that the gross motor task performed with HapticMASTER may have greatly contributed to the development of physical fatigue due to the increased physical demand. In contrast, the fine motor task performed with SCRIPT passive orthosis may have mainly induced mental fatigue due to the increased mental demand required during the task.

### 3.4. Association of Changes in Fatigue Level With the Substantive EEG Feature Modulations

**Figure 12:**
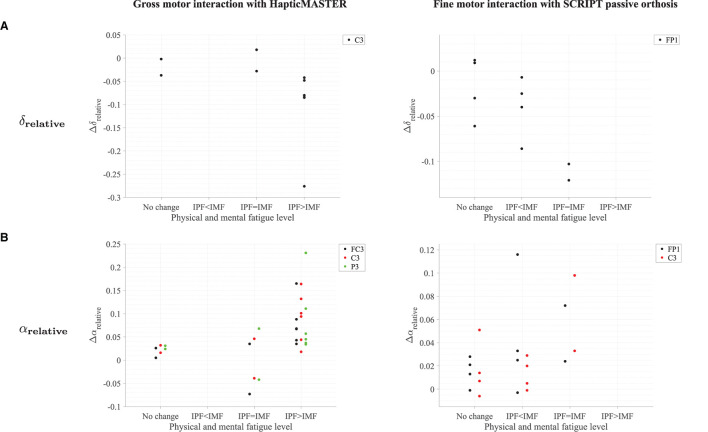
Comparison of the modulations in δ_relative_ and α_relative_ with the variations in fatigue levels following the **(A)** robot-mediated gross motor interaction with HapticMASTER and **(B)** robot-mediated fine motor interaction with SCRIPT passive orthosis. The Δ represents the difference in each EEG feature following the robot-mediated interactions (i.e., recovery - baseline). The “IPF” and “IMF” refers to the increase in physical and mental fatigue scores following the robot-mediated interactions, respectively. No change refers to no increase or a decrease in both fatigue levels.

Most participants who reported an increase in physical fatigue following the robot-mediated gross motor interaction also showed a greater increase in α_relative_ on FC3, C3, and P3 electrodes, (θ + α)/β and α/β on the C3 electrode in comparison to the participants who reported no change or reduction in physical fatigue. Similarly, a greater decrease in δ_relative_ on the C3 electrode was also found in most participants who experienced increased physical fatigue. Therefore, the above findings show that the significant changes in δ_relative_, α_relative_, (θ + α)/β and α/β around the motor cortex are likely related to the physical fatigue accumulated following the gross motor task. All six participants who reported increased mental fatigue following the robot-mediated fine motor interaction showed a decrease in δ_relative_ on the FP1 electrode. Five participants also showed an increase in α_relative_ on FP1 and C3 electrodes, and four participants showed an increase in θ_relative_ on C4. Therefore, the modulations in EEG spectral features around the prefrontal cortex presumably reflect increased mental fatigue following the robot-mediated fine movements. Figure 12 shows the association between the modulations in δ_relative_ and α_relative_ and the variations in subjective measures of physical and mental fatigue levels following the robot-mediated gross and fine motor tasks.

## 4. Discussion

This preliminary experiment investigated cortical-related changes associated with fatigue in upper limb robot-mediated gross and fine motor interactions. The findings of this experiment indicate that it is possible to monitor fatigue induced by robot-mediated interactions using EEG spectral features, which can have further utility for robot-mediated post-stroke therapy.

The most prominent finding was a significant increase in the α_relative_ following both the robot-mediated gross and fine motor interactions. It is known that α activity is most commonly visible during relaxed conditions and decreased attention levels. Also, in drowsy but wakeful states when increased efforts are taken to maintain the level of attention and alertness, increased α activity is visible (Klimesch, 1999). In contrast, when an individual is in an alert state, suppression of α activity is visible. Task-related desynchronisation, which leads to a decrease in α activity, can be interpreted as an electrophysiological correlate of increased activation of the cortical areas (excited neural structures) that produce motor behaviour or process sensory or cognitive information (Pfurtscheller et al., 1996; Pfurtscheller, 1997). Therefore, the increased α_relative_ following the robot-mediated interactions may reflect decreased cortical activity and a reduced capacity for information processing in the underlying cortical regions due to fatigue. This finding is in agreement with the findings of previous fatigue studies (Eoh et al., 2005; Barwick et al., 2012; Zhao et al., 2012; Fan et al., 2015; Zou et al., 2015). Thus, we suggest that the observed modulations in α_relative_ presumably reflect the changes in an individual's fatigue level following upper limb robot-mediated interactions. Furthermore, the above inference was also supported by the participants' feedback on the changes in their physical and mental fatigue levels after the assigned task; thereby suggesting that α_relative_ is a reliable EEG-based fatigue index that can be used to monitor fatigue accumulated during human-robot interactions.

Topographical differences found in the prominent EEG spectral features indicate that the brain regions most affected by fatigue may depend on the physical and mental workload associated with the task and the differences in the usage of the proximal and distal upper arm. In the gross motor interaction, participants were instructed to move the HapticMASTER robot arm in a linear trajectory to reach the two target points visible in the virtual reality environment. In a visually guided reaching task, the sensory system extracts spatial information about the target, and a movement plan is created and executed by the motor cortex (Sabes, 2000; Gevins and Smith, 2007). The premotor cortex, primary somatosensory cortex, and posterior parietal cortex integrate motor and sensory information for planning and coordinating complex movements. Also, HapticMASTER is an end-effector based robot, and the proximal upper limbs (arm and shoulder) are predominantly used when moving the robot arm between target points during the gross motor task. Therefore, the significant rise in α_relative_ found at FC3, C3, and P3 electrode locations presumably reflects the inhibition of premotor cortex, primary somatosensory cortex, and posterior parietal cortex due to physical fatigue accumulated during the arm reach/return task. A previous study has also shown that the upper limb reaching tasks performed using the HapticMASTER induced muscle fatigue (Thacham Poyil et al., 2020a,b). Conversely, in the fine motor task, participants were expected to perform hand open/close gestures only when a fish was near the seashell in the virtual environment. Therefore, the fine motor task required more sustained attention and decision-making than the gross motor task. Laureiro-Martínez et al. (2014) also found that a stronger activation in the frontopolar cortex is associated with higher decision-making efficiency. In addition, active movements consisting of repetitive opening and closing of the hand are shown to activate the contralateral primary sensorimotor cortex (Guzzetta et al., 2007). Therefore, the increased α_relative_ over FP1 and C3 electrodes following the repetitive fine movements appear to reflect an altered decision-making efficiency of an individual, in addition to the deactivation in the motor cortex associated with fatigue. The topographical variations in α_relative_ were also supported by the participants' feedback on their fatigue level after each interaction. The greater changes in α_relative_ following the gross motor task were also associated with a greater increase in physical fatigue. In contrast, the greater changes in α_relative_ following the fine motor task were associated with a greater increase in mental fatigue or an equal increase in both physical and mental fatigue levels.

It has been established in the literature that EEG activity shifts from high frequencies toward slower waves with the progression of fatigue; thus, the ratio between low-frequency and high-frequency power can also be considered as a reliable measure of fatigue (Eoh et al., 2005; Jap et al., 2009). This experiment found significant differences only in (θ + α)/β and α/β on the C3 electrode following the physically fatiguing gross motor task. These findings were also supported by the participants' feedback on their fatigue level. Furthermore, there were no significant differences in the power ratios due to the fine motor task. Although the significant changes on the C3 electrode were only visible for α_relative_, a slight increase in θ_relative_ and a decrease in β_relative_ were also found after the gross motor task. Therefore, the findings suggest that gross motor interaction increased the low-frequency activities while suppressing the high-frequency activities on the C3 electrode, which may have caused the significant increase of (θ + α)/β and α/β. Eoh et al. (2005); Jap et al. (2009); Chen et al. (2013); Fan et al. (2015) also reported a significant rise in both (θ + α)/β and α/β with fatigue.

The suppression in δ_relative_ following the robot-mediated interactions is contrary to some previous studies that have suggested a statistically significant increase or no significant difference in δ activities due to fatigue (Lal and Craig, 2002; Craig et al., 2012; Zhao et al., 2012). However, a non-significant reduction in δ_relative_ around all brain regions after a simulated driving task was reported in Zhao et al. (2012). In this experiment, a significant decrease in δ_relative_ was found on the C3 electrode following the gross motor task and on the FP1 electrode following the fine motor task. Most participants who reported an increase in their physical fatigue level after the robot-mediated gross motor task also have experienced a decrease in δ_relative_ on the C3 electrode. Similarly, all participants who reported an increase in their mental fatigue level following the robot-mediated fine motor task also showed a decrease in δ_relative_. Therefore, the subjective measures of fatigue level support the suppression in δ_relative_ due to fatigue build-up and the topographical variations found in the two tasks. The methodological differences of the previous studies could explain these discrepancies as these studies were based on vehicle driving tasks, whereas our experiment was focused on gross and fine motor tasks in a human-robot interaction scenario. Harmony et al. (1996) proposed that increased attention to internal processing (i.e., “internal concentration”) during mental tasks might cause an increase in the delta activity. In order to accurately perform the two tasks in this experiment, higher concentration and attention levels are essential. Therefore, the reduction in δ_relative_ associated with the robotic interactions may suggest deficient inhibitory control and information-processing mechanisms. This finding, while preliminary, suggests that fatigue may have negatively affected an individual's attention and internal concentration levels. Therefore, δ_relative_ could also be used as an EEG-based measure of fatigue in robot-mediated interactions.

The ipsilateral primary somatosensory cortex is also shown to increase its level of activation during prolonged sustained and intermittent sub-maximal muscle contractions to compensate for fatigue (Liu et al., 2003). In this experiment, a significant change in the C4 electrode was visible only for θ_relative_ following the fine motor task. Theta oscillations in EEG have shown to be prominent during cognitive processing that requires higher mental effort and is positively related to task difficulty (Gevins et al., 1997). Barwick et al. (2012), Cheng and Hsu (2011), and Zhao et al. (2012) also reported an increase in θ_relative_ due to fatigue build-up. Therefore, the rise in θ_relative_ on C4 may reflect the fatigue-related changes in the ipsilateral brain activation caused by the fine motor task.

The spatial precision of the EEG recordings taken in this experiment was limited since the EEG data acquisition system could only support eight electrode locations. Furthermore, only a limited number of participants were tested, and each participant interacted with only one robotic interface. Therefore, future research should examine more electrode locations and consider a cross-over study design with a higher sample size where each participant is exposed to both fatiguing robotic interactions.

## 5. Conclusion

This paper investigates the modulations in EEG spectral features associated with fatigue induced by robot-mediated upper limb gross and fine motor interactions. It was found that the fatigue induced by the gross movements mostly altered the EEG activity around the central and parietal brain regions, whereas the fine movements mostly altered the EEG activity around the frontopolar and central brain regions. These regional differences in significant EEG spectral features are most likely due to the differences in the nature of the task (fine/gross motor and distal/proximal upper limb) that may have differently altered the physical and mental fatigue level of an individual. We have shown that EEG correlates of fatigue progressed during robot-mediated interactions are specific to the physical or cognitive nature of the task performed using the proximal or distal upper limb. Further studies will explore whether the specificity is due to the difference in the motor skills considered (fine/gross motor) or the usage of upper limbs (distal/proximal upper limb). Given that fatigue during robot-mediated therapy can be estimated via EEG spectral features, we believe that the findings could potentially be used to moderate the level of fatigue during post-stroke rehabilitation, acknowledging that stroke patients are more likely to be fatigued than healthy individuals. Moreover, it would be possible to derive more personalised robot-mediated post-stroke rehabilitation regimes that would utilise the individual fatigue levels as a tool to increase the efficacy of upper limb robot-mediated rehabilitation.

## Data Availability Statement

The datasets presented in this article are not readily available because ethical consent to share the data publicly alongside publications was not sought at data collection, and therefore it is not possible to share the data. Requests to access the datasets should be directed to Farshid Amirabdollahian, f.amirabdollahian2@herts.ac.uk.

## Ethics Statement

The studies involving human participants were reviewed and approved by Science and Technology Ethics Committee with Delegated Authority of the University of Hertfordshire. The patients/participants provided their written informed consent to participate in this study.

## Author Contributions

UD carried out the data collection, analysis, and wrote the first draft of the manuscript. FA and VS obtained the research funding and supervised the research. All authors contributed to the conception and design of the experiment, manuscript revision, and approved the submitted version.

## Funding

This work was supported by the Ph.D studentship awarded to UD from the University of Hertfordshire, United Kingdom.

## Conflict of Interest

The authors declare that the research was conducted in the absence of any commercial or financial relationships that could be construed as a potential conflict of interest.

## Publisher's Note

All claims expressed in this article are solely those of the authors and do not necessarily represent those of their affiliated organizations, or those of the publisher, the editors and the reviewers. Any product that may be evaluated in this article, or claim that may be made by its manufacturer, is not guaranteed or endorsed by the publisher.

## Abbreviations

α_relative_: relative alpha band power
β_relative_: relative beta band power
δ_relative_: relative delta band power
θ_relative_: relative theta band power.
